# P-2234. Real World Impact of Metagenomic Next-Generation Sequencing Compared to Standard Infectious Diagnostic Workup

**DOI:** 10.1093/ofid/ofae631.2387

**Published:** 2025-01-29

**Authors:** Taylor Ackling, Helen Lee, Hannah H Nam

**Affiliations:** UC Irvine Medical Center, Long Beach, California; UCI Medical Center, Orange, California; University of Irvine - California, Orange, California

## Abstract

**Background:**

Metagenomic next-generation sequencing (mNGS) is a new, promising diagnostic tool for infectious diseases (ID). Of these mNGS tests, cell-free DNA (cfDNA) sequencing is one of the first commercially available and most common send-out tests by academic medical centers in the US. Unlike standard microbiology and serology diagnostic tests, cfDNA utilizes blood samples to measure plasma microbial cell-free DNA to detect over 1,000 pathogens. However, there is significant cost associated with the testing. Currently, there is a lack of a standardized approach for how to interpret cfDNA results with limited publications clearly defining its clinical impact and utility.

**Methods:**

We conducted a retrospective, descriptive study in an academic medical center of patients for whom cfDNA test was performed between January 2021 and September 2023 to determine its clinical impact and utility. The primary outcome of this study is the clinical impact of the cfDNA test categorized as positive, negative, indeterminate, or no impact. Positive impact is defined as leading to a new or earlier diagnosis compared to standard infectious workup, or escalation/de-escalation to appropriate antimicrobial agents. Negative impact is defined as leading to unnecessary treatment or additional diagnostic testing. Indeterminate impact is defined as patient mortality before test results. Secondary outcomes include disease-state indications for cfDNA testing. Clinical impact was adjudicated by an internal ID expert.

**Results:**

There was a total of 150 cfDNA tests included amongst 121 patients during the study period. Pneumonia (52%) was the leading indication for testing, followed by febrile neutropenia (16%), endovascular infections (9.3%), and fevers of unknown origin (9.3%). Based on preliminary analysis, 25/150 (16.7%) resulted in a positive impact, 1/150 (0.67%) resulted in a negative impact, and 110/150 (72%) had no impact on diagnosis or antimicrobial use. 14/150 (9.3%) tests were indeterminate. Of the cfDNA tests resulting in positive impact, 65.3% led to the initiation of new antibiotics.

**Conclusion:**

cfDNA testing may be useful in detecting new pathogens not found on standard workup, however, we found that it did not have a clinical impact in most cases in which it was utilized.

**Disclosures:**

All Authors: No reported disclosures

